# Molecular pathophysiology of chronic kidney disease–mineral and bone disorder: Focus on the fibroblast growth factor 23–Klotho axis and bone turnover dynamics

**DOI:** 10.1113/EP092401

**Published:** 2025-02-27

**Authors:** Alief Waitupu, Laras Pratiwi, Henry Sutanto, Djoko Santoso, Decsa Medika Hertanto

**Affiliations:** ^1^ Internal Medicine Study Program, Department of Internal Medicine, Faculty of Medicine Universitas Airlangga Surabaya Indonesia; ^2^ Department of Internal Medicine Dr Soetomo General Academic Hospital Surabaya Indonesia; ^3^ Division of Nephrology and Hypertension, Department of Internal Medicine, Faculty of Medicine Universitas Airlangga Surabaya Indonesia

**Keywords:** bone turnover, chronic kidney disease, FGF23, Klotho, mineral and bone disorder

## Abstract

Chronic kidney disease–mineral and bone disorder (CKD‐MBD) is a major complication of chronic kidney disease (CKD), characterized by disruptions in mineral metabolism, abnormal bone turnover and vascular calcification, which collectively increase the risk of fractures and cardiovascular disease. This review examines the molecular mechanisms underlying CKD‐MBD, with a particular focus on the fibroblast growth factor 23 (FGF23)–Klotho axis – a key regulator of phosphate balance, vitamin D activation and parathyroid hormone secretion. In CKD, elevated FGF23 levels and reduced Klotho expression contribute to mineral homeostasis disturbances and bone abnormalities. The dysregulation of this pathway plays a central role in CKD‐MBD pathophysiology and its associated complications. Emerging therapies, such as anti‐FGF23 antibodies and recombinant Klotho, hold promise for modulating FGF23 activity and restoring mineral balance. This review highlights the importance of individualized treatment strategies based on bone turnover patterns and FGF23–Klotho axis dysfunction. Advancing our understanding of these molecular mechanisms will aid in the development of more effective diagnostic tools and therapeutic interventions to improve CKD‐MBD outcomes.

## Abstract (highlights)

- **What is the topic of this review?**
  This review explores the molecular mechanisms driving bone turnover dynamics in CKD‐MBD, with a primary focus on the FGF23–Klotho axis, a critical regulator of phosphate balance, vitamin D activation and parathyroid hormone secretion.
- **What advances does it highlight?**
  The pathophysiology of bone turnover dynamics in CKD‐MBD is complex and interrelated, with multiple molecular and cellular determinants involved. This highlights the potential of emerging therapies targeting this multifaceted pathway, including anti‐FGF23 antibodies and recombinant Klotho, as well as PTH analogues, activin A receptor inhibitors and anti‐sclerostin antibodies.

## 1 INTRODUCTION

Chronic kidney disease–mineral and bone disorder (CKD‐MBD) is a systemic complication of advanced chronic kidney disease (CKD), affecting nearly all individuals with CKD stages 4 and 5 (Melamed et al., 2016). Characterized by disruptions in mineral metabolism, skeletal integrity and vascular health, CKD‐MBD significantly impacts patient well‐being and clinical outcomes (Bacchetta et al., 2021; Hruska & Seifert, 2013; Luo & Chen, 2020). A major challenge in CKD‐MBD management is addressing bone turnover abnormalities, which range from high‐turnover to low‐turnover states (Shah et al., 2024). These variations complicate both diagnosis – often requiring invasive bone biopsy – and treatment, as interventions must be carefully tailored to avoid exacerbating bone fragility and vascular calcification. Over‐suppression of parathyroid hormone (PTH) in high‐turnover disease, for example, can inadvertently push patients into a low‐turnover state, worsening skeletal and cardiovascular complications (Aguilar et al., 2023). A deeper understanding of CKD‐MBD's molecular mechanisms, particularly the fibroblast growth factor 23 (FGF23)–Klotho pathway, is essential for improving patient outcomes. As a key regulator of mineral metabolism, this pathway governs phosphate and calcium homeostasis while directly modulating bone turnover. This review examines the interplay between bone turnover abnormalities and the FGF23–Klotho axis in CKD‐MBD pathophysiology. Given its emerging therapeutic potential, targeting this pathway could offer new strategies for optimizing CKD‐MBD management.

## 2 PATHOPHYSIOLOGICAL OVERVIEW OF CKD‐MBD

**FIGURE 1:**
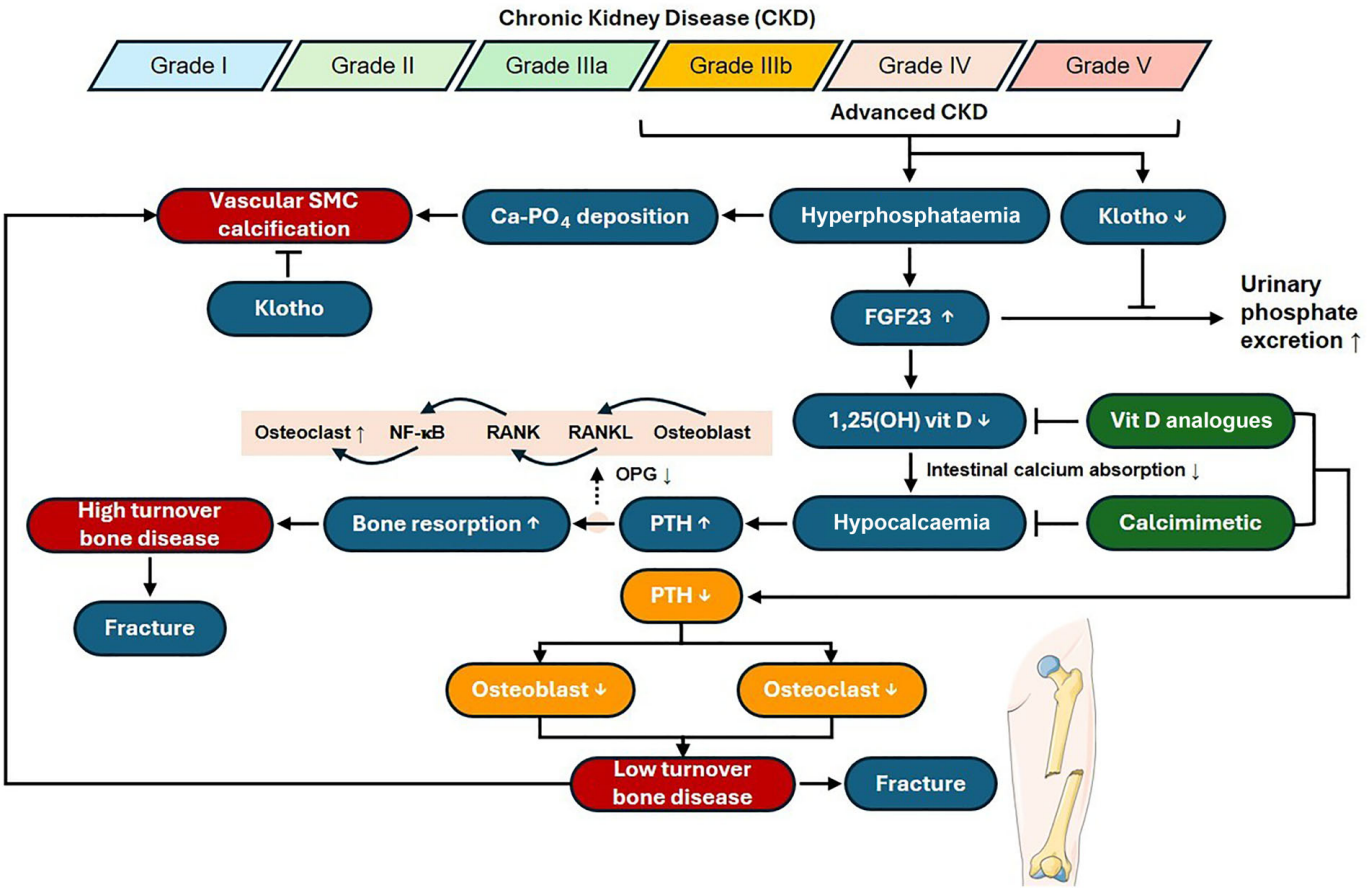
Basic pathophysiology of bone turnover disorders and vascular calcification in chronic kidney disease (CKD). As CKD advances, imbalances in calcium, phosphate, parathyroid hormone (PTH), vitamin D, and other factors such as fibroblast growth factor 23 (FGF23) and Klotho lead to significant complications. Key processes include vascular smooth muscle cell (SMC) calcification, calcium phosphate (Ca‐PO_4_) crystal deposition and disrupted bone remodelling, resulting in high or low turnover bone disease. The figure shows the pathways of bone resorption and the roles of osteoblasts and osteoclasts, regulated by the RANK/RANKL/NF‐κB system. The CKD grades were based on KDIGO (Stevens et al., 2024).

As CKD advances, patients frequently develop a range of mineral and bone abnormalities that define CKD‐MBD (Figure 1). Among the earliest and most significant changes is hyperphosphataemia, which results from the kidneys' diminished ability to excrete phosphate. Phosphate retention increases as glomerular filtration rate declines, leading to elevated serum phosphate levels. Hyperphosphataemia stimulates the secretion of FGF23, a hormone produced by osteocytes to increase phosphate excretion and suppress the production of 1,25‐dihydroxyvitamin D (i.e., active vitamin D). However, in CKD, the efficacy of FGF23 is diminished due to renal impairment, causing phosphate to accumulate further and disrupt normal mineral balance. Chronic phosphate retention directly contributes to vascular calcification and bone abnormalities, both of which are central complications of CKD‐MBD (Bacchetta et al., 2021; Shah et al., 2024). Another critical element in CKD‐MBD pathophysiology is hypocalcaemia, often arising from a combination of phosphate retention and vitamin D deficiency. The decline in active vitamin D production due to both kidney dysfunction and FGF23 activity reduces intestinal calcium absorption, resulting in low serum calcium levels. In response, the parathyroid glands increase PTH secretion, a compensatory mechanism aimed at mobilizing calcium from bones to restore serum levels, a process known as secondary hyperparathyroidism (Goyal et al., 2024; Rout & Jialal, 2024). Over time, prolonged elevations in PTH lead to high‐turnover bone disease, characterized by increased bone resorption and structural weakening. Although secondary hyperparathyroidism initially serves to counterbalance hypocalcaemia, its chronic effects drive pathological bone changes, contributing to the increased fracture risk observed in CKD patients (Bacchetta et al., 2021).

Vascular calcification is a hallmark of CKD‐MBD and a significant contributor to the heightened cardiovascular mortality among CKD patients (Hernandes et al., 2017; Izzo et al., 2024). Hyperphosphataemia and high serum calcium phosphate product levels promote the deposition of calcium phosphate crystals in vascular smooth muscle cells, leading to calcification of blood vessels. High phosphate levels induce a transformation in vascular smooth muscle cells, converting them to osteoblast‐like cells capable of depositing bone‐like minerals in the vessel walls. Subsequently, they begin to produce bone matrix proteins like alkaline phosphatase, collagen and osteocalcin, which facilitate calcium phosphate crystal deposition. Additionally, low bone turnover states, particularly adynamic bone disease (ABD), limit the skeletal system's ability to buffer excess calcium and phosphate, further exacerbating vascular calcification (Locatelli et al., 2002). Another factor that contributes to this calcification process is the decrease of calcification inhibitors. The deficiency of Klotho, a co‐receptor of FGF23 that has protective effects against calcification, accelerates the calcification process in CKD (Hu et al., 2011). In addition, healthy tissue normally contains inhibitors of calcification, such as matrix Gla protein (MGP), pyrophosphate and fetuin‐A, which prevent crystal deposition by inhibiting the nucleation and growth of calcium phosphate crystals (Azpiazu et al., 2018; Izzo et al., 2024). In CKD, levels of these inhibitors are often reduced or functionally impaired due to uraemic toxins, inflammation and oxidative stress. For instance, pyrophosphate is a potent inhibitor of calcification, but in CKD, its levels may drop due to both decreased synthesis and increased degradation. This loss of inhibitory control allows calcium phosphate crystals to form more easily and accumulate in tissues (Azpiazu et al., 2018). Vascular calcification is commonly seen in both large and medium‐sized arteries. Specifically, calcification frequently affects the aorta, coronary arteries and peripheral arteries. Unlike typical atherosclerotic calcification, which primarily involves the intimal layer of the arteries, vascular calcification in CKD‐MBD often occurs in the medial layer (i.e., medial calcification, also known as Monckeberg's arteriosclerosis) (Izzo et al., 2024). Vascular calcification is not merely an incidental finding in CKD‐MBD but rather a progressive condition that increases arterial stiffness and contributes directly to the elevated cardiovascular morbidity and mortality in CKD patients by increasing the risk of cardiovascular complications such as left ventricular hypertrophy, heart failure and myocardial infarction (Izzo et al., 2024; McCullough et al., 2008).

Wnt signalling also plays a crucial role in CKD‐MBD by influencing bone turnover and mineral metabolism (Hruska et al., 2017). In a healthy individual, Wnt proteins bind to receptors on osteoblasts and their precursors, activating the canonical Wnt/β‐catenin pathway, which allows β‐catenin to accumulate in the cell and move into the nucleus. There, β‐catenin influences gene transcription essential for osteoblast differentiation, proliferation and function, ultimately promoting bone formation and mineralization. In CKD, however, the balance within the Wnt signalling pathway is disturbed largely due to an increase in Wnt antagonists – specifically, proteins like sclerostin and Dickkopf‐1 (DKK1). Sclerostin is primarily produced by osteocytes and acts as an inhibitor of Wnt signalling by binding to the LRP5/6 receptors on osteoblasts, thereby preventing Wnt proteins from initiating the pathway. Similarly, DKK1 blocks Wnt signalling by binding to these receptors, diminishing Wnt/β‐catenin pathway activity. Elevated levels of these antagonists in CKD patients lead to a significant reduction in Wnt pathway activation, which limits osteoblast differentiation and function. This reduction in osteoblast activity results in decreased bone formation and contributes to conditions like ABD. The increased expression of sclerostin and DKK1 in CKD may be influenced by several factors, including altered PTH levels, inflammatory cytokines, and uraemic toxins, all of which are elevated in advanced CKD. For instance, while PTH normally stimulates bone turnover, its dysregulation in CKD can further drive imbalances in bone remodelling by affecting Wnt signalling directly or indirectly. Together, these factors exacerbate bone metabolism abnormalities in CKD patients, contributing to the development of CKD‐MBD (Bisson et al., 2018; Massy & Drueke, 2017; Zhang et al., 2023).

## 3 CLASSIFICATION OF BONE TURNOVER IN CKD‐MBD

Bone turnover abnormalities are a defining characteristic of CKD‐MBD and vary widely among affected patients. These abnormalities can be classified into high‐turnover bone disease, low‐turnover bone disease and normal bone turnover states, each associated with distinct skeletal and vascular outcomes (Table 1). The high‐ and low‐turnover forms of CKD‐MBD pose particular challenges in management, as they carry unique risks of bone fragility, fracture and calcification. High‐turnover bone disease, commonly referred to as osteitis fibrosa cystica (i.e., brown tumour), is typically driven by elevated PTH levels resulting from secondary hyperparathyroidism. In high‐turnover disease, osteoclast activity increases, leading to rapid bone breakdown, while osteoblasts attempt to compensate by increasing bone formation. This cycle of accelerated turnover results in structurally weakened bone that is prone to fractures and bone pain. Additionally, the abnormal bone remodelling associated with high‐turnover disease can lead to fibrous tissue formation and cyst‐like spaces within bones, further contributing to skeletal instability (Naji Rad et al., 2024).

**TABLE 1:** Bone turnover dynamics in CKD‐MBD.

Aspect	High‐turnover bone disease (osteitis fibrosa cystica)	Low‐turnover bone disease (adynamic bone disease)	Normal/intermediate turnover bone disease
Definition	High bone resorption and formation due to excess PTH from secondary hyperparathyroidism	Low bone remodelling activity due to suppressed PTH, often from treatment	Relatively balanced bone remodelling seen in early CKD stages
Epidemiology	More common in advanced CKD, particularly stages 4 and 5	Common in patients treated with high doses of calcimimetics or vitamin D analogues	Seen in early CKD stages (1–3), not yet fully dysregulated
Molecular distinctions	Elevated PTH stimulates excessive osteoclast and osteoblast activity	Over‐suppressed PTH leads to low osteoclast and osteoblast activity	Balanced PTH and FGF23 levels maintain bone turnover
FGF23 levels	Elevated, often increases with advanced CKD	Elevated but with limited impact due to suppressed bone turnover	Mildly elevated, compensating for kidney function decline
Klotho levels	Low, exacerbates phosphate retention and oxidative stress	Low, further suppresses osteoblast and osteoclast function	Moderate, adequate to support FGF23 function temporarily
Symptoms	Bone pain, muscle weakness, fatigue	Often asymptomatic until fractures occur	May be asymptomatic; mild bone or muscle discomfort in some cases
Signs	Increased alkaline phosphatase, elevated serum phosphate, decreased serum calcium	Normal or low PTH, low alkaline phosphatase, normal serum calcium and phosphate	Relatively normal serum markers; early signs of phosphate imbalance
Diagnosis	Diagnosis based on high PTH, FGF23, bone biopsy showing increased osteoclastic activity	Low PTH, bone biopsy showing minimal bone remodelling activity	Moderate PTH and FGF23; bone biopsy generally not required
Management	Calcimimetics, phosphate binders, vitamin D analogues to reduce PTH levels	Reduce calcimimetics or vitamin D analogues to allow normal bone turnover	Dietary phosphate management, monitoring to prevent progression
Complications	Fractures, skeletal deformities, cardiovascular disease from vascular calcification	Increased risk of fractures, high risk of vascular calcification due to low phosphate buffering	Potential to progress to high or low turnover disease if untreated
Prognosis	Poor if untreated, higher risk of fractures and cardiovascular complications	Guarded; increased risk of fractures and vascular complications if untreated	Good if monitored; potential to maintain bone health with early intervention

Low‐turnover bone disease (Table 1), for instance ABD, is characterized by suppressed bone remodelling due to low PTH levels. This condition is often associated with the over‐suppression of PTH from treatments like vitamin D analogues or calcimimetics. While these therapies can be effective in reducing PTH and preventing high turnover, excessive suppression can lead to a state where osteoblast and osteoclast activities are markedly reduced. In ABD, the lack of normal bone turnover impairs the bone's ability to repair microdamage, leading to increased fragility and fracture risk. Furthermore, low‐turnover bone disease is associated with a higher risk of vascular calcification, as the bone is less capable of buffering excess calcium and phosphate, promoting their deposition in vascular tissues instead (Sista & Arum, 2016). Another example of low‐turnover bone disease is osteomalacia. It develops when bone formation and resorption are both reduced, but it specifically involves a failure in the mineralization process rather than solely a reduction in cellular activity. Osteomalacia is characterized by the accumulation of unmineralized bone matrix, or osteoid, due to insufficient calcium and phosphate being deposited in the bone. This mineralization defect leads to weakened bones that are more susceptible to fractures and deformities (Eknoyan et al., 2003; Zaimi & Grapsa, 2024). The differences between ABD and osteomalacia are discussed in Table 2.

**TABLE 2:** Comparison between adynamic bone disease and osteomalacia.

Aspect	Adynamic bone disease (ABD)	Osteomalacia
Definition	Low‐turnover bone disease characterized by reduced bone formation and resorption without defective mineralization	Low‐turnover bone disease marked by defective mineralization and accumulation of unmineralized osteoid
Primary pathology	Suppression of osteoblastic and osteoclastic activity; minimal bone remodelling	Defective mineralization due to vitamin D deficiency, hypocalcaemia, or aluminium toxicity
Bone cell activity	Low osteoblast and osteoclast activity	Osteoblasts may be present but unable to effectively mineralize the osteoid
Histological features	Minimal bone formation with thin trabeculae and low osteoblast/osteoclast surface; normal mineralization	Excess unmineralized osteoid with wide osteoid seams; impaired mineralization
Bone turnover	Very low turnover, with minimal remodelling activity	Low turnover with impaired mineralization, leading to accumulation of soft, unmineralized bone
Mineralization status	Normal mineralization, but with reduced bone renewal and adaptation to stress	Defective mineralization, leading to soft, weakened bones
Calcium and phosphate handling	Normal serum calcium and phosphate levels, often managed by calcium‐based binders or vitamin D analogues	Hypocalcaemia and possibly hypophosphataemia due to low calcitriol levels, leading to impaired mineralization
Serum PTH levels	Low to suppressed due to over‐suppression by vitamin D analogues and calcium binders	Elevated if hypocalcaemia persists as a compensatory response, though not always sufficient to improve mineralization
Alkaline phosphatase levels	Typically low or within normal range, reflecting low bone formation rates	Often elevated, indicating impaired mineralization and osteoid accumulation
Common symptoms	Usually asymptomatic; fractures may occur due to brittle, poorly remodelled bone	Bone pain, muscle weakness, and deformities; increased fracture risk due to soft bone
Risk factors	Aggressive PTH suppression, calcium‐based phosphate binders, high vitamin D analogue doses	Vitamin D deficiency, aluminium exposure (historically), low calcitriol levels
Typical sites of bone impact	Trabecular and cortical bone; overall reduction in bone adaptability and remodelling	Weight‐bearing bones, leading to deformities in long bones, ribs, and pelvis
Imaging features	Reduced bone density without deformities; may show low bone volume	Looser's zones (pseudo fractures) due to unmineralized osteoid; diffuse osteopenia
Treatment strategy	Increase mild PTH stimulation by adjusting vitamin D and phosphate binder use; avoid over‐suppression of PTH	Correct vitamin D deficiency with calcitriol or analogues; avoid aluminium exposure; support mineralization
Response to vitamin D therapy	May worsen ABD if PTH suppression is too aggressive	Positive response, as vitamin D can correct calcitriol deficiency and improve mineralization
Long‐term complications	Fractures due to brittle bone structure, cardiovascular calcifications due to CKD‐MBD	Bone pain, deformities, fractures; impaired mobility and increased fracture risk

## 4 MOLECULAR MECHANISMS OF BONE TURNOVER DYSREGULATION IN CKD‐MBD

### 4.1 FGF23–Klotho–activin A axis and its role in bone turnover

In healthy individuals, FGF23 acts to maintain phosphate balance by promoting renal phosphate excretion and suppressing calcitriol synthesis through the reduction of 1α‐hydroxylase activity (Jüppner, 2011). However, in CKD, the kidneys’ reduced ability to excrete phosphate leads to excessive FGF23 production, resulting in elevated serum FGF23 levels (Lima et al., 2023). This overproduction of FGF23 impairs calcitriol synthesis, which reduces intestinal calcium absorption and leads to hypocalcaemia. The low calcium levels stimulate PTH secretion, resulting in secondary hyperparathyroidism – a key driver of high‐turnover bone disease in CKD‐MBD (Jüppner, 2011). The effects of FGF23 in CKD are further complicated by a concurrent decline in Klotho expression. Klotho is a protein that serves as an essential co‐receptor for FGF23 in renal tubules, enabling FGF23's signalling in the kidney to enhance phosphate excretion. As CKD progresses, Klotho levels diminish, impairing FGF23's ability to facilitate phosphate excretion effectively. This decline in Klotho contributes to the accumulation of serum phosphate and exacerbates bone‐mineral imbalances. Without sufficient Klotho, FGF23 is unable to achieve its regulatory effects fully, leading to further phosphate retention and promoting disturbances in calcium and PTH homeostasis. This imbalance fosters a vicious cycle where elevated FGF23 and low Klotho perpetuate the dysregulation of bone turnover, affecting both high‐turnover and low‐turnover bone diseases (Bacchetta et al., 2021; Kaludjerovic et al., 2017; Razzaque, 2009). Elevated FGF23 levels have been associated with both high and low bone turnover states, as the hormone disrupts the intricate feedback loops that regulate PTH and vitamin D. In high‐turnover bone disease, excessive FGF23 contributes to secondary hyperparathyroidism by limiting calcitriol synthesis, which prevents adequate calcium absorption and triggers continuous PTH release. In contrast, in low bone turnover, elevated FGF23, alongside suppressed PTH from aggressive therapy, results in reduced bone remodelling activity (Nagata et al., 2011). Nevertheless, a study involving end‐stage renal disease patients demonstrated that high bone turnover was associated with very high FGF23 levels, while low bone turnover was observed with somewhat lower FGF23 (Lima et al., 2014).

Activin A, a member of the transforming growth factor (TGF)‐β superfamily, is elevated in CKD and contributes to the pathophysiology of CKD‐MBD (Nordholm et al., 2023; Shankar et al., 2024). It signals through its receptor, a type II receptor serine/threonine kinase (ActRIIA), which activates downstream Smad signalling pathways. This activation drives inflammation, fibrosis and the suppression of osteoblast activity, thereby impairing bone formation while promoting bone resorption (Kundra et al., 2024; Sugatani, 2018). Consequently, activin A exacerbates bone turnover abnormalities in CKD‐MBD, further destabilizing skeletal integrity. Beyond its effects on bone, activin A also negatively impacts vascular health by promoting vascular smooth muscle cell calcification, increasing the risk of cardiovascular complications in CKD patients (Esposito et al., 2021). Its harmful effects are amplified by the concurrent decline in Klotho levels, as Klotho normally acts as a protective regulator by inhibiting activin A receptor activation. In CKD, elevated activin A can suppress Klotho expression directly or indirectly, creating a self‐reinforcing cycle of metabolic dysregulation. Without sufficient Klotho, tissues become more susceptible to activin A's deleterious effects, leading to increased bone fragility and vascular calcification (Agapova et al., 2016; Hruska et al., 2017). This complex interplay between activin A, FGF23, Klotho, PTH and vitamin D underscores the intricate molecular disruptions underlying CKD‐MBD.

### 4.2 Pathophysiology of high‐turnover bone disease

High‐turnover bone disease is characterized by accelerated bone resorption and formation, driven primarily by secondary hyperparathyroidism (Figure 1). Over time, this compensatory hypersecretion of PTH causes high bone turnover, wherein osteoclast activity is significantly increased, leading to excessive bone resorption (Hu et al., 2022). Elevated PTH stimulates osteoblasts on the bone surface to produce and release receptor activator of nuclear factor kappa‐B ligand (RANKL), a key mediator in osteoclast genesis. RANKL binds to its receptor, RANK, located on osteoclast precursors, activating the nuclear factor kappa‐light‐chain‐enhancer of activated B cells (NF‐κB) pathway. This signalling promotes the differentiation of precursor cells into mature osteoclasts (Aguilar et al., 2023; Cafiero et al., 2018). Additionally, PTH reduces the expression of osteoprotegerin (OPG), a decoy receptor that would otherwise bind to RANKL and inhibit its interaction with RANK. The decreased OPG/RANKL ratio under high PTH conditions thus enhances osteoclast recruitment and activation, facilitating an environment primed for accelerated bone resorption (Huang et al., 2004). Once activated, osteoclasts adhere to the bone matrix and release enzymes, such as cathepsin K and matrix metalloproteinases, which degrade the organic components of bone, along with hydrochloric acid, which dissolves the mineralized matrix, releasing calcium and phosphate back into circulation (Delaissé et al., 2003). This process is further amplified by PTH's direct effects on osteoblasts, which produce cyclic adenosine monophosphate (cAMP) and activate protein kinase A (PKA) signalling, enhancing the transcription of RANKL and other pro‐resorptive factors (Aguilar et al., 2023; Kondo et al., 2002). Moreover, PTH influences production of interleukin‐6, a cytokine that supports osteoclast differentiation and survival, thereby sustaining high levels of osteoclast activity (de la Mata et al., 1995; Grey et al., 1999). The cumulative effect of these molecular interactions is excessive bone resorption, which undermines bone integrity despite increased bone turnover. Although new bone is formed in response to PTH‐driven turnover, the rapid remodelling does not allow for proper mineralization, resulting in weakened bone structure. This high‐turnover state not only predisposes patients to fractures but also contributes to mineral imbalances and further exacerbates CKD‐MBD complications, creating a cycle of bone loss and remodelling dysregulation (Aguilar et al., 2023).

The impact of high‐turnover bone disease is profound, as it induces excessive bone remodelling that disrupts bone architecture. While bone resorption is accelerated, bone formation also increases as osteoblasts attempt to compensate for bone loss. However, this rapid cycle of bone breakdown and formation results in poor bone quality and density despite high bone volume, rendering bones structurally weak and prone to fractures. Additionally, the excessive activity of osteoclasts and osteoblasts can lead to bone lesions and fibrotic changes, further impairing bone integrity. The continuous remodelling process in high‐turnover bone disease fails to produce well‐mineralized bone, increasing the risk of skeletal complications and pain in CKD patients (Shah et al., 2024).

### 4.3 Pathophysiology of low‐turnover bone disease (adynamic bone disease)

Low‐turnover bone disease, or ABD, is characterized by markedly reduced bone remodelling activity due to excessive suppression of PTH. In CKD patients, treatments such as calcimimetics and vitamin D analogues, commonly used to control secondary hyperparathyroidism, can significantly lower PTH levels (Figure 1). Although this suppression reduces the risk of high‐turnover bone disease, it can also impair the activities of osteoblasts and osteoclasts, the cells responsible for bone formation and resorption (Couttenye et al., 1999; Shah et al., 2024). Reduced PTH levels lead to decreased mobilization of calcium and phosphate from bone, exacerbating mineral imbalances and contributing to a low‐turnover state where bone remains largely inactive. PTH plays a central role in bone remodelling by stimulating osteoblast activity directly and, indirectly, osteoclast formation through signalling mediators like RANKL (Huang et al., 2004; Xiong et al., 2014). In low‐PTH states, these pathways are suppressed, leading to decreased bone turnover. Osteoblasts rely on PTH for activation via the PTH/parathyroid hormone‐related protein (PTHrP) receptor pathway, which stimulates cAMP and downstream PKA signalling. Reduced PTH levels lead to decreased expression of RANKL and increased expression of OPG, both of which contribute to reduced osteoclast differentiation and activity. Without sufficient RANKL and with elevated OPG levels, osteoclast precursor cells receive inadequate signalling to mature, leading to a reduction in osteoclast activity and, consequently, bone resorption (Datta & Abou‐Samra, 2009; Huang et al., 2004; Martin, 2005). Additionally, low PTH reduces the expression of other anabolic factors such as insulin‐like growth factor 1 and bone morphogenetic proteins, which are critical for osteoblast function and bone matrix production (Khan et al., 2016; Tahimic et al., 2013). As a result, both bone formation and resorption are diminished, leading to a state of low bone turnover.

PTH resistance (or hyporesponsiveness) is another key factor contributing to low turnover bone disease in CKD. Normally, PTH plays a crucial role in regulating bone turnover by stimulating osteoblasts and osteoclasts, promoting both bone formation and resorption to maintain mineral balance (Silva & Bilezikian, 2015). However, in CKD, PTH resistance develops due to impaired PTH receptor signalling, reduced receptor expression on target cells, and altered intracellular pathways within bone cells. CKD causes the reduction of expression of PTH receptors (PTH1R) on target cells, particularly in bone and kidney tissue. Uraemic toxins that accumulate as kidney function declines are known to suppress PTH receptor gene expression, leading to fewer available receptors on the cell surface. This downregulation reduces the cells’ ability to respond to PTH (receptor desensitization), even when PTH levels are elevated (Bover et al., 2021). Additionally, there is evidence of disrupted cAMP and PKA signalling in bone cells, reducing the effectiveness of PTH. Elevated phosphate levels and metabolic acidosis in CKD further alter these pathways, impairing the PTH receptor's ability to properly signal within target cells. Next, CKD is associated with chronic inflammation and oxidative stress, which are both known to interfere with receptor signalling in various tissues, including bone. Oxidative stress leads to the production of reactive oxygen species, which can damage cellular components, including PTH receptors and associated signalling proteins. Inflammation further contributes by increasing pro‐inflammatory cytokines that can inhibit PTH signalling pathways, compounding the effects of PTH resistance. Collectively, these combined effects – downregulation of PTH receptors, uraemic toxin accumulation, intracellular signalling disruption, oxidative stress, inflammation, and elevated phosphate and FGF23 levels – result in an impaired PTH receptor signalling system in CKD (Bover et al., 2021; Evenepoel et al., 2016). As a result, despite high circulating PTH levels, this resistance dampens the response of osteoblasts and osteoclasts to PTH, leading to a substantial reduction in bone turnover. The resulting ABD is marked by low osteoblast and osteoclast activity, decreased bone formation, and a rigid, brittle bone structure. Furthermore, this low turnover state contributes to mineral dysregulation in CKD, as it impairs the bone's capacity to buffer calcium and phosphate, aggravating the already disturbed mineral metabolism in CKD‐MBD. PTH resistance in CKD thus not only leads to fragile bones but also exacerbates the broader complications of mineral imbalance (Iwasaki et al., 2006).

The impact of low‐turnover bone disease is significant, as it compromises bone resilience and increases fracture susceptibility despite normal or even elevated bone density. The lack of regular bone remodelling means that microfractures accumulate over time, leaving bones structurally fragile and more prone to fractures under stress. Additionally, the inactive bone turnover associated with ABD decreases the bone's ability to buffer excess calcium and phosphate. This impaired buffering capacity contributes to the increased risk of vascular calcification commonly observed in CKD, as excess calcium and phosphate are deposited in vascular tissues rather than being sequestered in bone (Brandenburg & Floege, 2008; Frazão & Martins, 2009). Consequently, ABD not only heightens fracture risk but also indirectly promotes vascular calcification, exacerbating cardiovascular complications in CKD patients.

## 5 SEX DIFFERENCES IN CKD‐MBD

The differences in sex hormones, bone remodelling rates and vascular calcification processes contribute to distinct pathophysiological presentations of CKD‐MBD in men and women. The FGF23–Klotho axis, which plays a pivotal role in mineral metabolism and bone turnover, exhibits sex‐dependent regulation, influencing phosphate homeostasis, calcium dynamics, and skeletal integrity differently in males and females (Tsuchiya et al., 2015). One key factor in the sex‐dependent nature of CKD‐MBD is the influence of oestrogen and testosterone on the FGF23–Klotho axis. Oestrogen has been shown to enhance Klotho expression, which may provide protective effects against phosphate retention and vascular calcification in premenopausal women. Conversely, testosterone influences bone resorption and formation, potentially leading to a higher incidence of secondary hyperparathyroidism in men with CKD‐MBD (Komaba, 2010). Additionally, postmenopausal women experience a decline in oestrogen levels, which has been associated with increased FGF23 secretion and reduced Klotho expression, contributing to an accelerated decline in bone health and increased vascular calcification risk (Kuro‐o, 2017). Sex‐based disparities in CKD‐MBD also extend to differences in bone turnover markers and the risk of fractures. Men with CKD generally exhibit higher bone turnover rates, with increased osteoclast activity and greater cortical bone loss, which predisposes them to fractures involving long bones. In contrast, women, particularly postmenopausal patients, demonstrate more pronounced trabecular bone deterioration due to oestrogen deficiency, leading to a higher incidence of vertebral fractures (Ribeiro et al., 2020). This disparity may be exacerbated by the differential regulation of FGF23, PTH and vitamin D metabolism between the sexes. Another critical aspect of sex‐dependent differences in CKD‐MBD lies in the impact of vascular calcification. Women with CKD often have higher FGF23 levels relative to men at equivalent stages of kidney dysfunction, which may contribute to an increased cardiovascular burden despite potential skeletal benefits (Jimbo & Shimosawa, 2014). Men, on the other hand, tend to have more pronounced vascular calcification due to higher serum phosphate levels and reduced Klotho expression, leading to greater arterial stiffness and cardiovascular morbidity (Yamada & Giachelli, 2017).

## 6 CLINICAL MANAGEMENTS OF BONE TURNOVER DYSREGULATION IN CKD‐MBD

### 6.1 Diagnostic approaches to identify bone turnover states

Accurate assessment of bone turnover states is essential for managing CKD‐MBD, as treatment strategies depend on understanding whether a patient's bone metabolism is in a high‐turnover, low‐turnover or intermediate state. Non‐invasive serum biomarkers, such as PTH, FGF23 and alkaline phosphatase, are widely used in clinical settings to estimate bone turnover and guide treatment decisions (Ginsberg & Ix, 2022; Waziri et al., 2019). Elevated PTH levels are often associated with high‐turnover bone disease, or osteitis fibrosa cystica, driven by secondary hyperparathyroidism. Conversely, low PTH levels, especially when actively suppressed by treatments like calcimimetics or vitamin D analogues, may indicate a low‐turnover state, or ABD, where bone remodelling is significantly reduced. Serum alkaline phosphatase, particularly bone‐specific alkaline phosphatase (BSAP), serves as an additional marker, as it reflects osteoblastic activity; elevated levels are often seen in high‐turnover states, while low or normal levels may suggest low‐turnover bone disease. Serum FGF23, which increases early in CKD to enhance phosphate excretion, also holds diagnostic value, as its levels rise progressively with advancing CKD and may indicate evolving mineral dysregulation (Hughes‐Austin et al., 2020; Vervloet et al., 2017).

While these biomarkers provide valuable insights, they have limitations and can sometimes yield overlapping results, making it challenging to differentiate turnover states precisely (Qi et al., 1995; Sardiwal et al., 2013). For instance, elevated PTH levels do not always confirm high‐turnover disease, as PTH responsiveness may be altered in CKD (Bover et al., 2021). Similarly, FGF23 and alkaline phosphatase levels may fluctuate with factors unrelated to bone turnover, such as inflammation and other systemic conditions common in CKD. FGF23, for instance, is elevated not only in response to phosphate retention in CKD but also due to inflammatory processes and iron deficiency, both of which are common in CKD patients. Inflammation, a frequent occurrence in CKD due to uraemic toxins and comorbidities, can independently stimulate FGF23 production, making it challenging to distinguish if elevated FGF23 is due to mineral imbalances or inflammatory activity. Additionally, iron deficiency, prevalent in CKD due to reduced erythropoiesis, stimulates FGF23 production through molecular pathways linked to iron‐regulatory proteins, further confounding its interpretation (Czaya & Faul, 2019). BSAP, a marker of osteoblastic activity and bone formation, can similarly be affected by non‐skeletal factors. In CKD, BSAP levels can be influenced by liver dysfunction, as BSAP is an isoenzyme of total alkaline phosphatase, which has both liver and bone origins. Liver disease, common in CKD patients due to overlapping risk factors, can therefore elevate total alkaline phosphatase levels, potentially skewing BSAP results (Lowe et al., 2024). Medications often used in CKD, such as phosphate binders, can also indirectly impact BSAP by altering mineral balance and PTH activity, affecting bone turnover indirectly (Udomkarnjananun et al., 2022). Together, these non‐bone‐related factors make it essential to consider the broader clinical context when interpreting FGF23 and BSAP levels in CKD‐MBD.

Dual‐energy X‐ray absorptiometry (DEXA or DXA) is a widely used imaging modality for measuring bone mineral density (BMD) and assessing fracture risk, but its applicability in diagnosing bone turnover diseases in CKD‐MBD is limited. While DEXA provides valuable information on BMD, it does not offer insights into bone turnover rates or bone quality, both of which are crucial for understanding CKD‐MBD. Bone turnover disorders in CKD‐MBD, such as high‐turnover osteitis fibrosa cystica and low‐turnover ABD, involve changes in the activity of osteoblasts and osteoclasts, which affect bone remodelling dynamics rather than density alone. As a result, patients with CKD‐MBD can have normal or even elevated BMD on DEXA scans but still suffer from poor bone quality and increased fracture risk due to abnormal turnover. Additionally, DEXA cannot differentiate between cortical and trabecular bone, which is relevant in CKD‐MBD as cortical bone loss is particularly prominent in these patients (Lloret et al., 2024; Malluche et al., 2013). For a more comprehensive evaluation of bone turnover, DEXA needs to be supplemented with other diagnostic tools, such as biochemical markers (e.g., PTH, BSAP) or advanced imaging techniques like high‐resolution peripheral quantitative computed tomography (HR‐pQCT), which can assess bone microarchitecture and turnover more directly.

Due to these limitations, bone biopsy remains the gold standard for diagnosing bone turnover states, as it allows for direct assessment of bone remodelling activity, mineralization and structural integrity. Bone biopsy can definitively identify high‐turnover bone lesions, fibrotic tissue in osteitis fibrosa cystica, and the lack of bone remodelling in ABD. However, the invasiveness of bone biopsy, along with associated procedural risks and costs, limits its practicality in routine clinical use, and it is generally reserved for cases where non‐invasive assessments are inconclusive or when specific diagnoses are critical to treatment planning (Carbonare et al., 2021). As a result, developing more refined, non‐invasive diagnostic tools and enhancing biomarker reliability remains a priority in CKD‐MBD management. The ideal approach would combine biomarkers with advanced imaging techniques or novel biochemical indicators that offer greater specificity for bone turnover states.

### 6.2 Therapeutic strategies based on bone turnover type

Effective management of CKD‐MBD requires targeted therapeutic strategies that address the specific bone turnover state of each patient, as the pathophysiology and risks associated with high‐turnover and low‐turnover bone disease differ significantly. For high‐turnover bone disease, therapies focus on reducing PTH levels to slow bone loss and mitigate the risk of fractures. Calcimimetics, such as cinacalcet, are commonly used to lower PTH by enhancing the sensitivity of the calcium‐sensing receptor on parathyroid cells, thereby suppressing PTH secretion (Melamed et al., 2016). By controlling PTH levels, calcimimetics can reduce excessive bone remodelling and help stabilize bone structure (Hou et al., 2022). In addition, phosphate binders are often prescribed to control hyperphosphataemia, a key driver of PTH elevation in CKD. By reducing serum phosphate levels, phosphate binders lower the stimulus for PTH release, helping to control secondary hyperparathyroidism (Sprague et al., 2021). Vitamin D analogues (e.g., calcitriol or paricalcitol) are also used to manage high‐turnover disease by directly suppressing PTH synthesis. However, vitamin D therapy must be carefully monitored, as excessive dosing can contribute to hypercalcaemia, potentially worsening vascular calcification risks in CKD patients (Zand & Kumar, 2017).

In contrast, the treatment of low‐turnover bone disease (i.e., ABD) requires a different approach, as the main issue is an over‐suppression of bone remodelling. Low‐turnover bone disease often results from excessive suppression of PTH, commonly due to prolonged use of calcimimetics or vitamin D analogues. In these cases, therapeutic strategies focus on adjusting or minimizing these medications to restore balanced bone remodelling activity (Sista & Arum, 2016). For patients with ABD, reducing or discontinuing calcimimetics and carefully tapering vitamin D therapy can help stimulate PTH release, encouraging osteoblastic and osteoclastic activity to re‐establish normal bone turnover. This cautious approach is essential, as restoring some degree of bone remodelling helps prevent the accumulation of microdamage and reduces fracture risk. Additionally, restoring bone activity supports phosphate buffering capacity, potentially reducing vascular calcification by allowing more phosphate to be incorporated into bone rather than deposited in vascular tissues. The need to tailor therapy based on bone turnover type underscores the complexity of CKD‐MBD management, as treatments beneficial for one turnover state may exacerbate risks in another. For example, while calcimimetics and phosphate binders are effective for high‐turnover disease, they may lead to or worsen low‐turnover states if not carefully adjusted. Hence, monitoring biomarkers like PTH, FGF23 and BSAP is crucial for guiding and adjusting treatment in real‐time.

### 6.3 Emerging therapies targeting FGF23–Klotho axis

As research into CKD‐MBD has elucidated the crucial role of the FGF23–Klotho axis in mineral metabolism and bone health, novel therapies targeting this pathway have emerged, offering promising strategies for restoring mineral balance and mitigating bone disease. The FGF23–Klotho axis is dysregulated in CKD due to persistently high FGF23 levels and declining Klotho expression, which together contribute to phosphate retention, impaired vitamin D activation, and secondary hyperparathyroidism – all of which drive the mineral and bone abnormalities characteristic of CKD‐MBD. To counter these effects, recombinant Klotho and anti‐FGF23 antibodies are being developed as potential therapies aimed at normalizing the FGF23–Klotho pathway. Recombinant Klotho therapy seeks to replenish Klotho levels, which naturally decrease as CKD progresses. Restoring Klotho expression has been shown to improve mineral homeostasis by enhancing phosphate excretion and increasing calcitriol production, thereby helping to prevent the onset of hyperphosphataemia, hypocalcaemia and secondary hyperparathyroidism. Additionally, Klotho has antioxidant and anti‐inflammatory effects, which could protect against the vascular calcification often seen in CKD‐MBD. Experimental animal studies in rodents suggest that recombinant Klotho can mitigate FGF23's pathological effects in CKD, reducing bone and cardiovascular complications. However, this therapy is still in early stages of development, and more research is needed to determine its safety, efficacy and optimal dosing in humans (Hu et al., 2017; Kanbay et al., 2024).

Anti‐FGF23 antibodies (e.g., KRN23 or burosumab) represent another innovative approach, designed to neutralize the excessive FGF23 levels that contribute to mineral and bone disturbances in CKD. By blocking FGF23, these antibodies prevent FGF23‐mediated suppression of calcitriol synthesis, thereby restoring vitamin D levels and improving calcium absorption. This increase in calcitriol could help control secondary hyperparathyroidism by reducing the stimulus for PTH release, ultimately normalizing bone turnover and promoting better bone health. In animal models (rats), anti‐FGF23 antibody therapy has shown promise in reversing some of the bone and mineral abnormalities associated with CKD‐MBD. However, this approach carries risks, as completely inhibiting FGF23 might lead to hyperphosphataemia if phosphate excretion is insufficient (Imel et al., 2019; Shalhoub et al., 2012). Currently, there are no clinical trials specifically targeting FGF23 inhibition in patients with CKD‐MBD. Most clinical research on FGF23 inhibition has focused on conditions like X‐linked hypophosphataemia (XLH). For instance, burosumab is currently approved for treating XLH (Brandi et al., 2022). Together, these emerging therapies targeting the FGF23–Klotho axis hold potential to address the root molecular causes of CKD‐MBD, potentially offering more precise control over bone and mineral metabolism.

Anti‐sclerostin antibodies (e.g., romosozumab and blosozumab) have emerged as promising therapeutic agents that can selectively inhibit sclerostin and thus boost Wnt signalling. By blocking sclerostin's binding to LRP5/6, these antibodies allow Wnt ligands to activate the Wnt/β‐catenin pathway, leading to increased osteoblast differentiation, survival and activity. This promotes new bone formation and improves bone density and strength. Anti‐sclerostin therapy could be particularly beneficial for CKD‐MBD patients by addressing the specific deficits in bone turnover associated with high sclerostin levels (Kohler et al., 2024; Mukaddam et al., 2021; Sista & Arum, 2016). Early clinical trials show positive outcomes on bone density, but long‐term studies are needed to fully understand the risks and benefits. In a phase 2 clinical trial, blosozumab treatment in postmenopausal women with low BMD resulted in substantial increases in spine and hip BMD. Specifically, the highest dose group experienced a 17.7% increase at the spine and a 6.2% increase at the total hip. Biochemical markers indicated a rapid rise in bone formation, with BSAP levels remaining elevated compared to placebo at study end. Mild injection site reactions were reported more frequently with blosozumab than placebo (Recker et al., 2015). Similarly, in a phase 2, multicentre clinical trial, romosozumab has been associated with increased BMD and bone formation in postmenopausal women with low bone mass. Romosozumab treatment led to significant gains in BMD at the lumbar spine, total hip and femoral neck, along with increased markers of bone formation and decreased markers of bone resorption (McClung et al., 2014). Nonetheless, while anti‐sclerostin antibodies hold promise, CKD‐MBD patients present unique clinical challenges. These patients often have altered mineral metabolism, including elevated phosphate levels and dysregulated PTH levels, which further complicate bone health. Currently, there are no clinical trials specifically investigating anti‐sclerostin therapies in patients with CKD‐MBD (Costa et al., 2015). Clinical trials are needed to carefully assess the effects of anti‐sclerostin antibodies specifically in CKD populations, as bone response and vascular calcification risk may differ from non‐CKD patients. Additionally, because sclerostin is involved in regulating vascular calcification and mineral balance, blocking it could have unintended effects on cardiovascular health (Cejka, 2021). Beyond anti‐sclerostin therapy, other Wnt modulators, including anti‐DKK1 antibodies, are being investigated to address bone turnover abnormalities in CKD‐MBD (Fang et al., 2014). Targeting both sclerostin and DKK1 might offer synergistic benefits, as these antagonists work through overlapping mechanisms (Evenepoel et al., 2015). Additionally, small molecule inhibitors and other biologics that fine‐tune Wnt signalling are under exploration to achieve a balance in bone formation and resorption without adverse effects on vascular health (Tran & Zheng, 2017).

Therapeutic strategies targeting the activin A receptor in CKD‐MBD also have promising potential to counteract the harmful effects of elevated activin A levels and restore a healthier balance in bone turnover and mineral metabolism. Blocking activin A receptor activity can help restore osteoblast function and bone formation, which are often suppressed in CKD‐MBD due to high activin A levels. These therapies may promote a healthier balance between bone resorption and formation, improving overall bone density and quality, which would help reduce fracture risk in CKD‐MBD patients. Moreover, targeting the activin A receptor may help prevent or reduce the vascular calcification process, which could lower the risk of cardiovascular complications (Coyne et al., 2019). Inhibiting this pathway may also help limit fibrosis and inflammation, potentially slowing CKD progression and reducing complications associated with chronic inflammation. By inhibiting the activin A receptor, it may be possible to mitigate mineral metabolism disruptions in CKD, helping restore a more balanced mineral profile. Clinically, activin A receptor inhibitors (e.g., sotatercept and rapafercept) could complement other CKD‐MBD therapies, such as phosphate binders, vitamin D analogues, and Wnt pathway modulators (e.g., anti‐sclerostin antibodies). Sotatercept, for instance, is a fusion protein that acts as a ligand trap, binding to activin A and other members of the TGF‐β superfamily, thereby preventing them from binding to their receptors and activating downstream signalling pathways (Agapova et al., 2016). By targeting multiple pathways involved in CKD‐MBD, a comprehensive approach may yield synergistic benefits, improving patient outcomes more effectively than single therapies alone.

PTH analogues play a complex yet promising role in the management of CKD‐MBD by modulating bone turnover and helping restore balance, though their use requires caution due to the risk of exacerbating hypercalcaemia or calcification in these patients. PTH analogues, such as teriparatide (PTH 1–34) and abaloparatide, are primarily anabolic agents that stimulate osteoblast activity, encouraging new bone formation. Teriparatide has been used in certain cases of ABD in CKD to increase bone formation and promote remodelling in patients with very low bone turnover. By activating the osteoblasts, teriparatide can help CKD patients who have become susceptible to fractures due to the lack of bone renewal associated with low turnover (Igarashi et al., 2023; Nishikawa et al., 2016; Sista & Arum, 2016). Abaloparatide is another PTH analogue with a similar anabolic effect, though it has a slightly different receptor binding profile that may reduce hypercalcaemia risk compared to teriparatide (Phipps et al., 2019; Sista & Arum, 2016).

## 7 SUMMARY

CKD‐MBD is characterized by mineral metabolism disturbances, abnormal bone turnover, vascular calcification, increasing fracture and cardiovascular risks. The FGF23–Klotho axis plays a central role, regulating phosphate excretion, vitamin D activation and PTH secretion. In CKD, elevated FGF23 and reduced Klotho impair phosphate homeostasis and bone remodelling, contributing to secondary hyperparathyroidism and skeletal fragility. Bone turnover abnormalities vary from high‐turnover disease, driven by excessive PTH, to low‐turnover states like adynamic bone disease, where suppressed bone remodelling increases vascular calcification risk. Additional molecular regulators, including activin A and Wnt signalling inhibitors like sclerostin, further disrupt bone and vascular health. Emerging therapies targeting the FGF23–Klotho axis, activin A receptors and sclerostin aim to restore mineral balance and improve bone health. Individualized treatment approaches are essential to optimizing outcomes based on bone turnover state and disease progression.

## AUTHOR CONTRIBUTIONS

Alief Waitupu, Laras Pratiwi, Henry Sutanto, Djoko Santoso, and Decsa Medika Hertanto contributed to the conceptual framework of the review. All authors searched and interpreted the literature. Henry Sutanto was responsible for conceptualizing and designing figures. All authors wrote and revised the manuscript. All authors approved the final version of this manuscript and agree to be accountable for all aspects of the work in ensuring that questions related to the accuracy or integrity of any part of the work are appropriately investigated and resolved. All persons designated as authors qualify for authorship, and all those who qualify for authorship are listed.

## CONFLICT OF INTEREST

None declared.

## FUNDING INFORMATION

No funding was received for this work.
